# Supplementary material for: Bridging classical and quantum interpretation of chemical state analysis by XPS/HAXPES to resolve short-range order in amorphous alumina films
Source: J Mater Chem A Mater. 2026 Mar 11;14(24):15364–85. doi: 10.1039/d5ta08586a (PMC12977175; doi:10.1039/d5ta08586a)
Supplement: TA-014-D5TA08586A-s001 [file TA-014-D5TA08586A-s001.pdf]

Supplementary Information (SI) for Journal of Materials Chemistry A.
This journal is © The Royal Society of Chemistry 2026

Supplementary Materials: Bridging classical and quantum interpretation of chemical state analysis by XPS/HAXPES to resolve short-range order in amorphous alumina films

Simon Gramatte[1,2,3,4], Xing Wang[5,6], Michael Alejandro Hernández Bertrán[7], Claudia Cancellieri[3], Giovanni Pizzi[5,6], Deborah Prezzi[7], Iurii Timrov[6], Olivier Politano[2], Ivo Utke[8], Lars P.H. Jeurgens[3], and Vladyslav Turlo[1,4,*]

[1]Laboratory for Advanced Materials Processing, Empa - Swiss Federal Laboratories for Materials Science and Technology, Feuerwerkerstrasse 39, 3602 Thun, Switzerland
[2]Laboratoire Interdisciplinaire Carnot de Bourgogne, UMR 6303 CNRS-Université de Bourgogne, 9 Avenue A. Savary, 21078 Dijon Cedex, France
[3]Laboratory for Joining Technologies and Corrosion, Empa - Swiss Federal Laboratories for Materials Science and Technology, Ueberlandstrasse 129, 8600 Duebendorf, Switzerland
[4]National Centre for Computational Design and Discovery of Novel Materials (MARVEL), Empa, Thun, Switzerland
[5]National Centre for Computational Design and Discovery of Novel Materials (MARVEL), 5232 Villigen PSI, Switzerland
[6]PSI Center for Scientific Computing, Theory, and Data, 5232 Villigen PSI, Switzerland
[7]Nanoscience Institute – National Research Council (CNR-NANO), I-41125 Modena, Italy
[8]Laboratory for Mechanics of Materials and Nanostructures, Empa - Swiss Federal Laboratories for Materials Science and Technology, Feuerwerkerstrasse 39, 3602 Thun, Switzerland
[*]Corresponding author: Vladyslav Turlo, vladyslav.turlo@empa.ch

February 9, 2026

S1. Experiment

S1.1. ALD of *am*-Al_2O_3

The ALD of amorphous alumina, as detailed in Ref. [1, 2], involves sequential exposure of the substrate to trimethylaluminum (TMA) and water (H_2O), resulting in the formation of a thin amorphous alumina film through a series of self-limiting surface reactions. As shown previously [3, 2], there is a strong correlation between the quantity of unreacted OH groups and the amorphous alumina film density; i.e., the amorphous alumina density increases with increasing process temperature, corresponding to a decrease in the H-impurity content and respective H/Al ratio.

Sapphire (α-Al_2O_3) and Bayerite (α-$Al(OH)_3$) were selected as *crystalline* oxide and hydroxide reference phase, respectively. To this end, a one-side polished α-Al_2O_3[0001] single crystal was purchased from CrysTec GmbH, Germany. The Bayerite hydroxide reference was prepared according to the procedure described in Ref. [1]. Bayerite is a trihydroxide with an H/Al ratio of 3 and marks the extreme upper limit of the H/Al spectrum; its density aligns closely with that of the amorphous

alumina grown at ambient temperature [1]. Sapphire is the most stable bulk alumina oxide and therefore represents the extreme lower end of the H/Al spectrum with a corresponding density of $\rho = 3.9$ g/cm^3. All Al cations in sapphire and bayerite are octahedrally coordinated by O and OH ligands, respectively. This makes them exemplary reference materials for the exploration of ligand polarizability effects, as discussed in the following.

Table S1: Experimentally determined values and estimated errors for the average densities, H/Al atomic ratios, and measured Auger parameters of the Al cations, for amorphous alumina films deposited by ALD, as well as for the crystalline oxide and trihydroxide reference phases, sapphire, and bayerite. See Ref. [1, 4, 5] for more details on the amorphous films and crystalline references. The corresponding densities and H/Al atomic ratios, as simulated in the present study, are also listed. A reference value for the Auger parameter of free Al gas of 1454 eV [6] was used to calculate the corresponding Auger parameter shifts, $\Delta\alpha_{\text{Al}}$: see [7]. The final error of the Auger parameter shifts $\Delta\alpha_{\text{Al}}$ of 0.2 eV, accounting for the experimental error α_{Al} of 0.1 eV [1] and assumes the same error for the Auger parameter of the free gas. Notably, the corresponding Auger parameter for oxygen does not show a correlation with the H content and has an average value of $\Delta\alpha_{\text{O}} \approx 1039.7\pm$ 0.3 eV, as reported in Ref. [1]. The value for Al (crystalline) is an unpublished value, that is necessary for later comparison.

Sample	Density [g/cm^3]		H/Al ratio		E_{b}^{2p} [eV]	$E_{\text{k}}^{\text{KL}_{23}\text{L}_{23}}$ [eV]	α_{Al} [eV]	$\Delta\alpha_{\text{Al}}$ [eV]	$E_{\text{B}}^{O,1s}$ [eV]
	Exp.	Sim.	Exp.	Sim.					
Bayerite	2.4 – 2.6	2.50	3	3	74.1	1386.9	1461.0	7.0	531.6
Amorphous (25°C)	2.39±0.12	2.29	2±0.05	2	74.7	1386.4	1461.1	7.1	531.7
Amorphous (50°C)	2.84±0.11	2.60	1.07±0.05	1	74.6	1387.0	1461.6	7.6	531.7
Amorphous (120°C)	3.05±0.06	2.99	0.19±0.01	0.2	74.7	1387.2	1461.9	7.9	531.8
Amorphous (150°C)	3.07±0.06	3.00	0.13±0.01	0.125	74.8	1387.2	1462.0	8	531.7
Amorphous (200°C)	3.04±0.05	3.01	0.06±0.01	0.0625	74.8	1387.6	1462.4	8.4	531.7
Sapphire	3.9 – 4.0	3.90	0	0	73.7	1388.0	1461.7	7.7	531.3
Al (crystalline)	2.7	2.77	0	0	72.7	1393.25	1465.0	12.0	

S1.2. Details on Atomistic Simulation Cells

Table S2: Overview of the computational supercells adopted in this work, reporting the structural phase, ALD process temperature, hydrogen-to-aluminum ratio, the counts of aluminum, oxygen, and hydrogen atoms, as well as the complete set of cell parameters a, b, c, α, β, and γ.

Sample	H/Al ratio	# Al	# O	# H	a [Å]	b [Å]	c [Å]	α [°]	β [°]	γ [°]
Sapphire	0.00	108	162	0	14.39	14.39	13.08	90.00	90.00	120.00
Bayerite	3.00	128	384	384	20.33	17.46	18.68	90.00	90.41	90.00
Amorphous (25°C)	2.00	128	320	256	19.97	16.74	18.60	90.18	91.03	90.38
Amorphous (50°C)	1.00	128	256	128	19.14	17.04	14.49	90.29	90.43	88.13
Amorphous (120°C)	0.20	128	205	26	16.89	14.45	15.53	89.21	89.78	88.55
Amorphous (150°C)	0.13	128	200	16	16.74	14.65	15.05	92.33	88.29	88.46
Amorphous (200°C)	0.06	128	196	8	16.58	14.00	15.26	89.90	92.28	88.93

S1.3. XPS/HAXPES Analysis

Combined XPS/HAXPES analysis was performed using a PHI Quantes spectrometer (ULVAC-PHI) equipped with a soft Al-Kα (1486.6 eV photon energy) and a hard Cr-Kα X-ray source (5414.7 eV photon energy). The linearity of the energy scale of the hemispherical analyzer (i.e., from 0 eV up to 5400 eV) was calibrated according to ISO 15472 by referencing the Au 4f7/2, Ag 3d5/2 and Cu 2p3/2 main peaks to the recommended binding energy E_{B} positions of 83.96 eV, 368.21 eV, and 932.62 eV, respectively (as measured in situ for the sputter-cleaned, high-purity metal references using both the soft and hard X-ray source). Charge neutralization during each measurement cycle

was accomplished by dual-beam charge neutralization, employing low-energy electron and Ar ion beams (1-V bias, 20-μA current). First, survey spectra were recorded from all samples with both the soft Al-Kα (@50 W) and the hard X-ray Cr-Kα source, as reported in Ref. [1]. Next, high-resolution spectra of the Al 2p, O 1s, and C 1s core levels, as well as of the O KLL Auger line, were measured with both X-ray sources using a power of 50 W, a step size of 0.1 eV, and a constant pass energy of 69 eV, resulting in a constant analysis area in the range of 200 - 280 μm^2. To enhance the signal from the interior of the oxide films and the bulk references, all XPS/HAXPES measurements were performed at a take-off angle of 90°between the sample surface and the center of the entry lens of the analyzer [8]. The Al KLL Auger transition can only be measured using Cr-Kα radiation (adopting the same measurement parameters as above). Notably, a pass energy of 69 eV was carefully selected, since it resulted in a similar energy resolution for both X-ray sources, as evidenced by a constant full-width-at-half-maximum of the Ag 3d5/2 peak of 0.9 eV for the XPS and HAXPES analysis. It is further noted that the probing depth of Al 2p photoelectrons excited with Al-Kα radiation is approximately the same as the probing depth of the Al KLL Auger electrons, thus ensuring chemical-state analysis at a near constant probing depth of about 9 nm [8].

Data analysis involved peak fitting of the Shirley-background corrected spectra using a single Gaussian-Lorentzian peak component, as fitted to the top of each peak studied (i.e., from approximately 0.7 of the maximum height of the respective peak) using the MultiPak 9.9 software provided by ULVAC-PHI. Since the soft and hard X-ray sources operate with different photon fluxes (and photon energies), their absolute binding energy scales need to be corrected for possible differences in electronic charging, as performed here by aligning the two sets of spectra (as recorded with both sources) to their respective adventitious C 1s peak at 284.7 eV. The correctness of this charge-correction procedure was confirmed by verifying that the photon-source-independent kinetic energy (KE) peak positions of the charge-corrected O KLL spectra from both X-ray sources were identical within ±0.1 eV. The measured (energy-scale-corrected) Al 2p and Al KLL spectra, as well as the corresponding Auger parameter shifts versus the experimental H/Al ratios (i.e. $\Delta\alpha$ with respect to Al gas) are shown in Figs. S1(a-c), respectively.

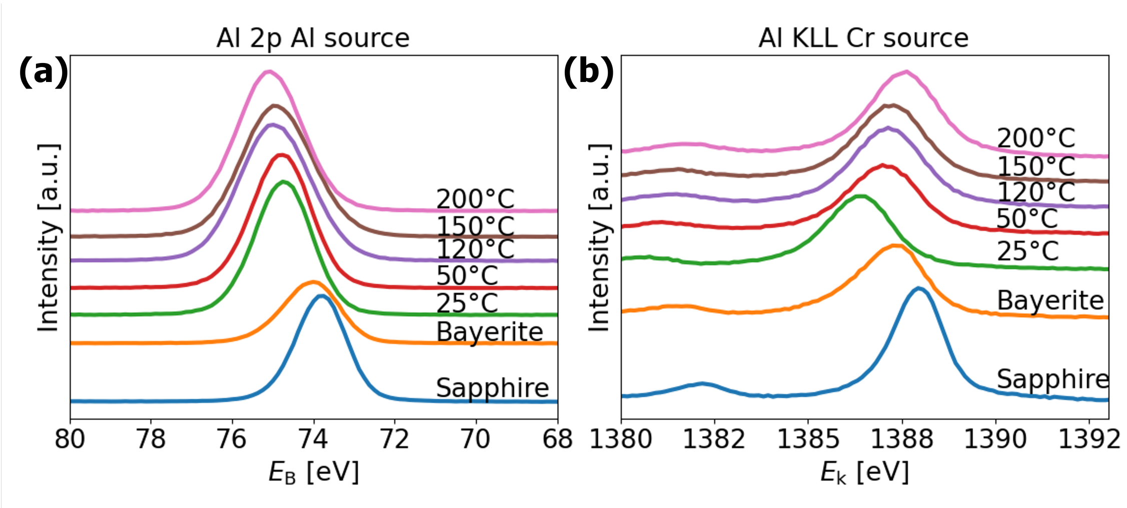

Figure S1: (a) Measured Al 2p spectra (with an inverted E_B scale by convention) and (b) Al KLL Auger spectra (with a KE scale) for all ALD and crystalline samples. The spectra's intensity is represented in arbitrary units (a.u.) and has been vertically stacked for enhanced distinguishability. The measured spectra were charge-corrected by aligning them to their adventitious C 1s peak at 284.7 eV (see Sec. S1.3).

The thus obtained values of Al 2p E_B and Al KLL KE will be further denoted as $E_\mathrm{b}^{2\mathrm{p}}$ and $E_\mathrm{k}^{\mathrm{KL_{23}L_{23}}}$, respectively: see Table S1. No clear correlation between $E_\mathrm{b}^{\mathrm{Al\ 2p}}$ and $E_\mathrm{k}^{\mathrm{Al\ KL_{23}L_{23}}}$ was found. However, a clear experimental correlation between the H/Al ratio and $\Delta\alpha_\mathrm{Al}$ is evidenced, which nicely confirms the specific sensitivity of the Auger parameter to final state relaxation effects (see Section 1). In particular, no experimental correlation was obtained between the corresponding Auger parameter for oxygen and the H content, giving an average value of $\Delta\alpha_\mathrm{O} = E_\mathrm{b}^{\mathrm{O1s}} + E_\mathrm{k}^{\mathrm{OKLL}} \approx$ 1039.7± 0.3 eV [1]. As discussed in Ref. [1], only the local chemical state of Al shows a clear trend

with increasing ALD deposition temperature (i.e., with decreasing H content). Therefore, in the following, we will focus only on modeling the Al Auger parameter shift with increasing H content in the amorphous alumina films.

S2. Coordination

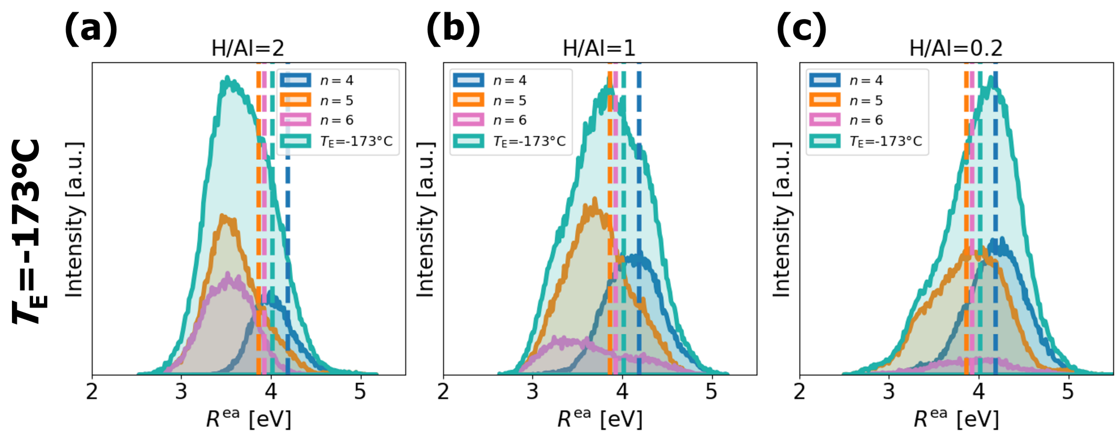

Figure S2: Analysis of the NNCS contributions to R^{ea} (for thermal equilibration at $T = -173$ °C), as based on the nearest-neighbor ligands count (i.e. $n = 4, 5, 6$) for amorphous polymorphs with (a) H/Al=2, (b) H/Al=1 and (c) H/Al=0.2. Dashed lines highlight the mean value of each individual distribution. Individual R^{ea} distributions originating from the 4-fold, 5-fold and 6-fold NNCs variants (independent of their O/OH ligand fraction).The corresponding R^{ea} distributions from each individual NNCS variant are displayed in Fig. 9 (a-c).

S3. Charge-density response to an O core hole in sapphire and bayerite

To assess why oxygen Auger parameters are more difficult to interpret in hydrogenated alumina, we analyze the valence charge-density response to an O full-core-hole (FCH) excitation in sapphire and bayerite. We compute the valence charge-density difference $\Delta\rho_v$ from ΔKS-DFT ground-state and FCH calculations following Sec. 4.1. The resulting redistribution patterns are shown as isosurfaces in Fig. S3 and quantified via the radially integrated charge difference $\Delta Q(r)$ in Fig. S4. In this way, we provide a direct, materials-specific comparison of how oxygen screening differs from the Al core-hole case, using the same analysis framework as in the main text.

Figure S3 highlights a qualitative contrast between Al and O core holes. For an Al core hole, screening is predominantly extra-atomic, i.e., charge accumulates near the ionized Al due to transfer from neighboring O ligands, consistent with a picture of screening dominated by charge flow from nearest neighbors. For an O core hole, the response is dominated by a strong local contraction/depletion pattern around the ionized oxygen, accompanied by polarization of surrounding atoms. This multi-center character is consistent with the partially delocalized, bonding nature of O-derived valence states. It complicates an interpretation in terms of localized dipoles or purely first-shell screening, especially when comparing oxides and hydroxides.

To quantify the spatial extent of the screening response, we evaluate $\Delta Q(r)$, as given in Sec. 4.1. As shown in Fig. S4, the compensating charge for an O core hole is largely supplied by charge already localized near the oxygen site itself, indicating a substantial intra-atomic screening contribution. At the same time, the curves reveal additional non-local polarization contributions at larger radii, consistent with the qualitative isosurface patterns in Fig. S3. In bayerite, O-H bonding provides an additional electronic reservoir that can be accessed by nearby oxygen atoms, further increasing the number of available screening channels and reinforcing the multi-center character of

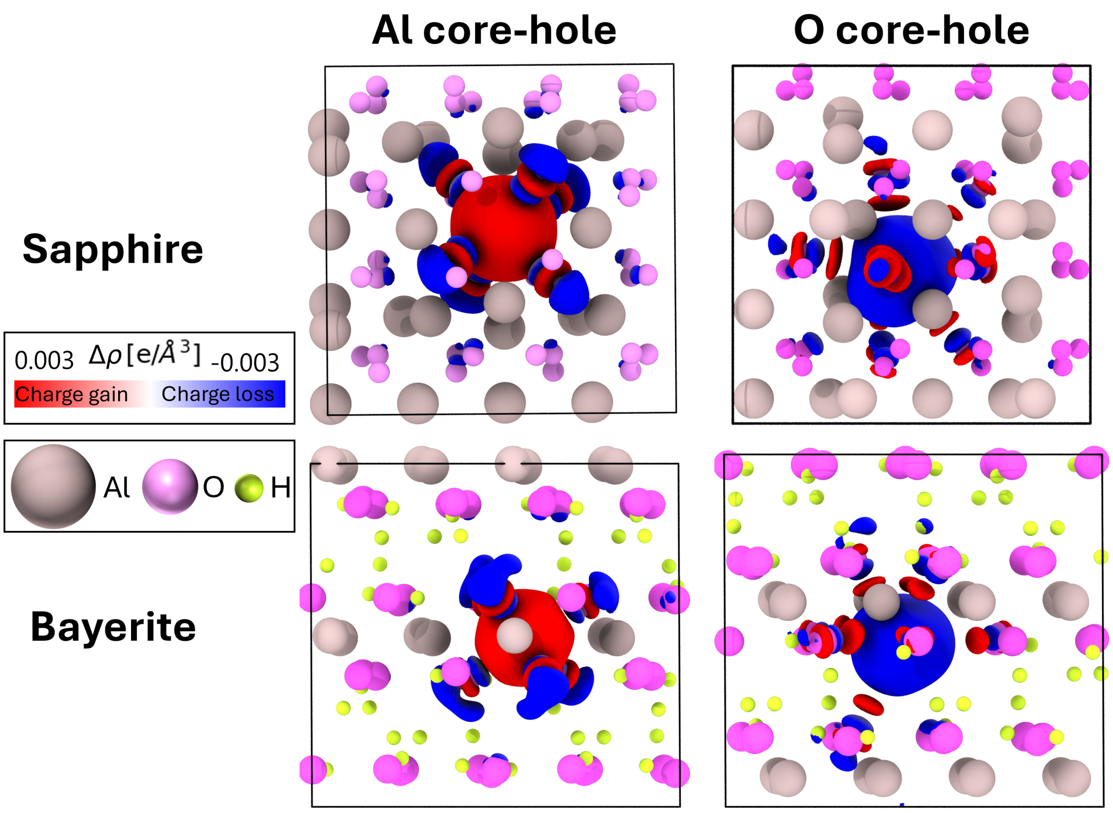

Figure S3: Valence charge-density differences $\Delta\rho_v$ for sapphire (top row) and bayerite (bottom row), shown as isosurfaces. Left column: Al core hole; right column: O core hole. Red indicates charge accumulation and blue indicates charge depletion induced by the core-hole excitation.

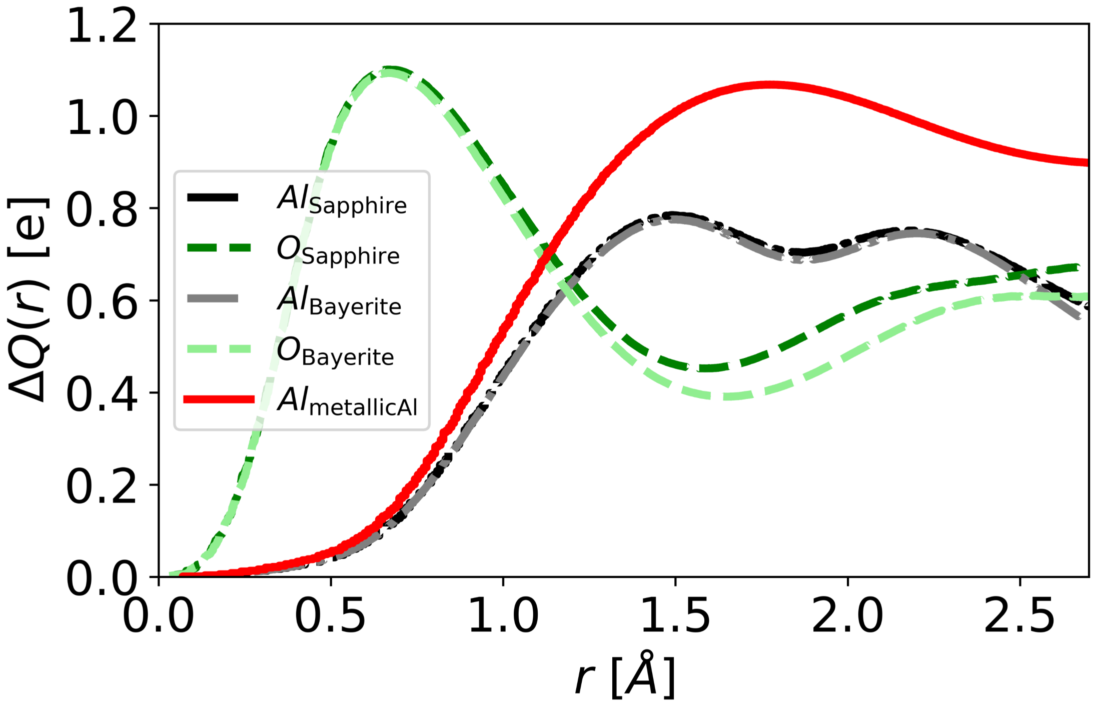

Figure S4: Radially integrated valence charge difference $\Delta Q(r)$ around the core-ionized site, computed from $\Delta\rho_{\mathrm{v}}$. Solid lines: Al core hole in sapphire (black) and bayerite (grey). Dashed lines: O core hole in sapphire (dark green) and bayerite (light green). Results for an Al core hole in metallic Al are included for reference.

the response. Overall, these results support our choice not to use oxygen Auger parameters for quantitative analysis in the present work and suggest that extensions of the electrostatic model should be addressed in follow-up work to capture such multi-center screening and polarization effects consistently.

S4. Building Blocks

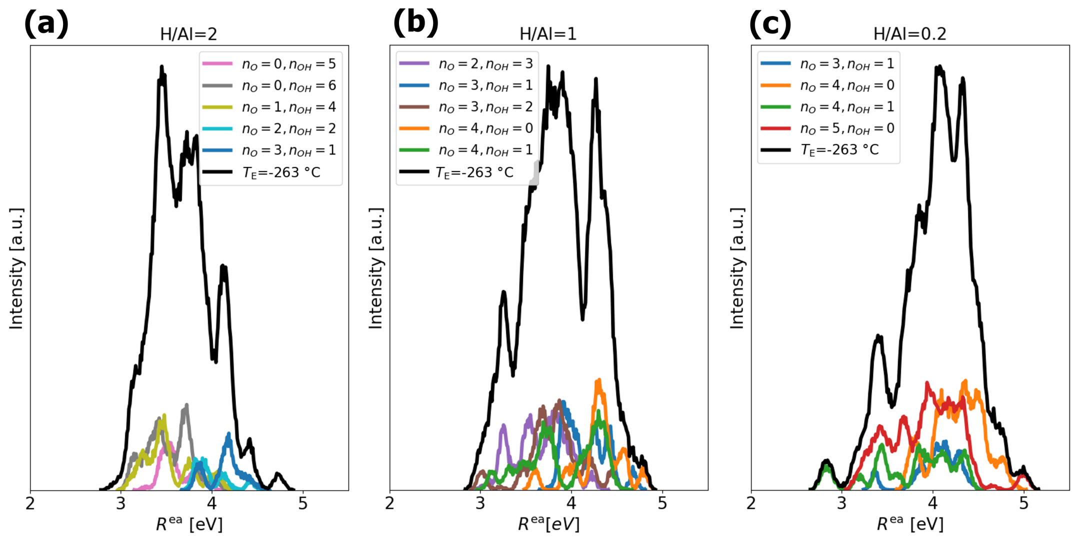

Figure S5: Figures (a), (b), and (c) present the full R^{ea} distribution at $T_E = -263°C$ for the respective H/Al ratios (2, 1, and 0.2), corresponding again to ALD temperatures of 25°C, 50°C, and 120°C. These figures also highlight the R^{ea} values for significant Al ligand groups, illustrating their individual contributions to the overall distribution.

For H/Al=2 (Fig. 9 (a)), the splitting into three distinct subpeaks of the full $T = -263°C$ R^{ea} distribution can each be correlated with specific n_O, n_{OH} groups. The first subpeak, featuring the lowest R^{ea}, predominantly originates from the $n_O = 0$, $n_{OH} = 6$ and $n_O = 1$, $n_{OH} = 4$ groups, while the third subpeak, represents higher R^{ea} values which are largely composed of the $n_O = 3$, $n_{OH} = 1$ group. The central peak does not distinctly correspond to specific groups but appears to be a composite of several contributions. For the H/Al=1 sample (Fig. 9 (b)), three major subpeaks can again be discerned. The lowest energy peak primarily originates from the group with the highest OH content, specifically $n_O = 2$, $n_{OH} = 3$. The middle subpeak, similar as for H/Al=2, consists of mixed contributions from several groups. The highest energy subpeaks are predominantly composed of the $n_O = 4$, $n_{OH} = 1$ and $n_O = 4$, $n_{OH} = 0$ groups, that have the most O ligands. For H/Al=0.2, (Fig. 9 (c)), a minor peak at low energy primarily consists of the $n_O = 4$, $n_{OH} = 1$ group, the principal group containing OH ligands. The remainder of the distribution largely emanates from the $n_O = 5$, $n_{OH} = 0$ and $n_O = 4$, $n_{OH} = 0$ groups. Overall, these distributions reveal that groups with a greater number of ligands exhibit slightly reduced R^{ea}. Furthermore, groups predominantly bonded with OH ligands tend to shift R^{ea} towards lower energies, whereas Al groups mainly coordinated with O ligands tend to shift R^{ea} towards higher energies, which should be attributed to the different ligand polarizabilities of O and OH.

S5. PFP Potential Validation

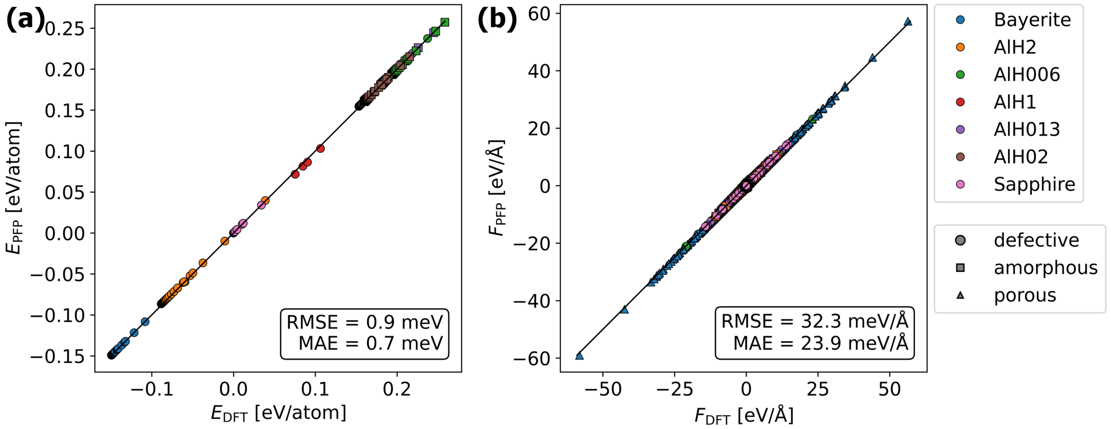

Figure S6: (a) Comparison of formation energies E computed using the PFP+D3 machine-learning potential versus DFT reference values across a diverse set of alumina and hydroxide-based structures, including amorphous, porous, and defective phases. (b) Comparison of force F predicted by the PFP+D3 potential against DFT-calculated forces for the same structural configurations. Both panels report the root mean square error (RMSE) and mean absolute error (MAE). Marker shapes distinguish structural types (amorphous, porous, defective), while marker colors indicate specific structure families. The black diagonal line indicates perfect agreement.

S5.1. Correction energies for the ΔKS method: The case of Al

The correction energies in Eq. 12 can be explicitly expressed as

$$\Delta E_{\mathrm{corr}} = \Delta E_{\mathrm{core}} - \delta, \tag{S1}$$

where ΔE_{core} accounts for the relaxation of the remaining core electrons in the presence of a core-hole and δ is an additional energy shift that one should add to compare directly with experimental data, mostly depending on the approximations to the exchange-correlation term [9]. ΔE_{core} is determined by performing an all-electron calculation on the target atom with and without the core-hole [10], allowing for the extraction of the core contribution to the total energy, usually not included in pseudopotential calculations. This term can be written as:

$$\Delta E_{\mathrm{core}} = E^{*}_{\mathrm{core}} - E^{0}_{\mathrm{core}} = E^{*}_{\mathrm{atom|total}} - E^{*}_{\mathrm{atom|val}} - (E^{0}_{\mathrm{atom|total}} - E^{0}_{\mathrm{atom|val}}), \tag{S2}$$

where $E^{*}_{\mathrm{atom|total}}$ ($E^{0}_{\mathrm{atom|total}}$) and $E^{*}_{\mathrm{atom|val}}$ ($E^{0}_{\mathrm{atom|val}}$) are extracted from the all-electron calculation performed during the core-hole (ground state) pseudopotential generation for the Al atom. In our case, ΔE_{core} amounts to 1518.4, 126.4, and 89.8 eV for the 1s, 2s, and 2p core levels, respectively.

The δ term is obtained by fitting a linear model (see Figure S7) using experimental and calculated E_{B} as

$$E_{\mathrm{B,calc}} = E_{\mathrm{B,exp}} + \delta. \tag{S3}$$

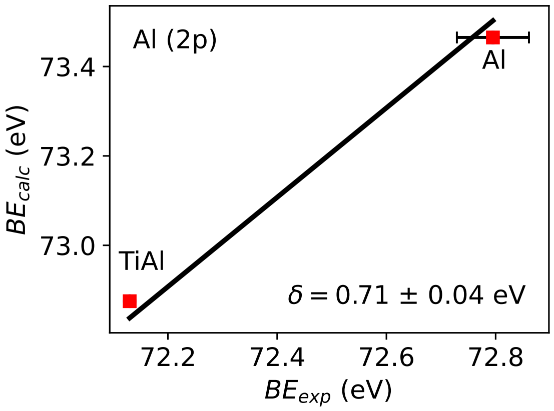

Figure S7: Calculated E_{B} compared to experimental values taken on our Al samples and from the NIST database [11], for both Al and TiAl. The error bar reflects the spread on the Al 2p E_{B} across the dataset, and the red points are placed at the mean value. TiAl was chosen as an additional reference for its metallic character, enabling accurate energy alignment within ΔKS, and its all-symmetrically equivalent Al atoms, eliminating ambiguities in assigning XPS contributions. The constant offset δ is the correction to the absolute $E_{\mathrm{B,calc}}$ corresponding to the PBE exchange-correlation functional [12].

In this procedure, the experimental E_{B} should correspond to systems where the reference energy level is well defined (i.e., accessible through DFT calculations), such as in molecules or metals.

S6. TEM image

A cross-sectional TEM image of a 42 nm thick amorphous Al_2O_3 film grown by ALD at 50°C is provided in Fig. S8. The sample was chosen to closely match the material and processing conditions considered in this work. The substrate consists of granular Pt grains (bright contrast) embedded in a carbonaceous matrix (dark contrast). Within the spatial resolution of the TEM measurement, the alumina layer appears laterally uniform and featureless at the nanometer scale. In particular, over the ∼1.5 nm length scale relevant for the periodic simulation cells used here, no obvious structural inhomogeneities or density modulations are discernible. While this observation does not remove the conceptual limitations associated with periodic repetition of a finite simulation cell, it is consistent with the assumption that simulation boxes below ∼2 nm provide a reasonable representation of the local amorphous environment for the quantities discussed in this study.

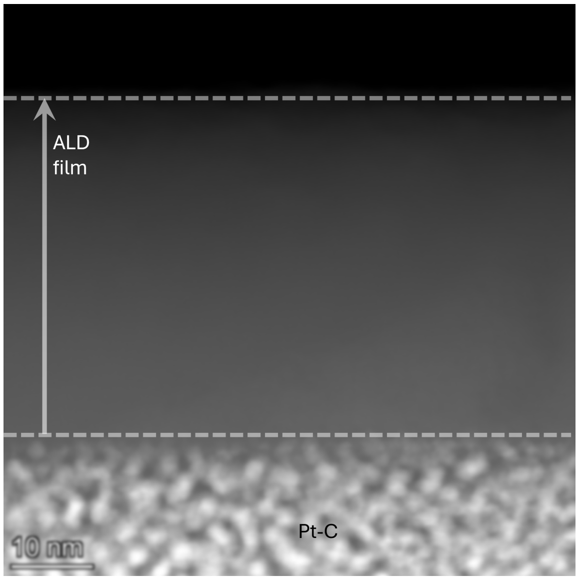

Figure S8: Cross-sectional TEM image of a 42 nm thick amorphous Al_2O_3 film grown by ALD at 50°C. The substrate shows granular Pt grains (bright contrast) embedded in a carbonaceous matrix (dark contrast). Within the spatial resolution of the measurement, the amorphous alumina layer appears laterally homogeneous.

References

[1] C. Cancellieri, S. Gramatte, O. Politano, L. Lapeyre, F. F. Klimashin, K. Mackosz, I. Utke, Z. Novotny, A. M. Müller, C. Vockenhuber, V. Turlo, and L. P. H. Jeurgens, "Effect of hydrogen on the chemical state, stoichiometry and density of amorphous Al2O3 films grown by thermal atomic layer deposition," *Surf. Interface Anal.*, vol. 56, no. 5, pp. 293–304, 2024.

[2] S. Gramatte, O. Politano, N. Jakse, C. Cancellieri, I. Utke, L. P. H. Jeurgens, and V. Turlo, "Unveiling hydrogen chemical states in supersaturated amorphous alumina via machine learning-driven atomistic modeling," *npj Comput. Mater.*, vol. 11, p. 170, jun 2025.

[3] C. Guerra-Nuñez, M. Döbeli, J. Michler, and I. Utke, "Reaction and Growth Mechanisms in Al2O3 deposited via Atomic Layer Deposition: Elucidating the Hydrogen Source," *Chem. Mater.*, vol. 29, no. 20, pp. 8690–8703, 2017.

[4] V. Palkar, D. Thapa, M. Multani, and S. Malghan, "Densification of nanostructured alumina assisted by rapid nucleation of α-alumina," *Mater. Lett.*, vol. 36, no. 5-6, pp. 235–239, 1998.

[5] F. Zigan, W. Joswig, and N. Burger, "Die Wasserstoffpositionen im Bayerit, Al(OH)3," *Zeitschrift für Krist. - Cryst. Mater.*, vol. 148, no. 3-4, pp. 255–274, 1978.

[6] F. Filippone and G. Moretti, "Oxide electronic polarizabilities and aluminum coordination at the outer surface of zeolites obtained by X-ray photoelectron spectroscopy," *Appl. Surf. Sci.*, vol. 135, no. 1-4, pp. 150–162, 1998.

[7] S. Gramatte, O. Politano, C. Cancellieri, I. Utke, L. P. H. Jeurgens, and V. Turlo, "Effect of hydrogen on the local chemical bonding states and structure of amorphous alumina by atomistic and electrostatic modeling of Auger parameter shifts," *arXiv*, 2024.

[8] S. Siol, J. Mann, J. Newman, T. Miyayama, K. Watanabe, P. Schmutz, C. Cancellieri, and L. P. H. Jeurgens, "Concepts for chemical state analysis at constant probing depth by lab-based XPS/HAXPES combining soft and hard X-ray sources," *Surf. Interface Anal.*, vol. 52, no. 12, pp. 802–810, 2020.

[9] M. Walter, M. Moseler, and L. Pastewka, "Offset-corrected Δ-kohn-sham scheme for semiempirical prediction of absolute x-ray photoelectron energies in molecules and solids," *Phys. Rev. B*, vol. 94, p. 041112, jul 2016.

[10] T. Mizoguchi, I. Tanaka, S.-P. Gao, and C. J. Pickard, "First-principles calculation of spectral features, chemical shift and absolute threshold of elnes and xanes using a plane wave pseudopotential method," *J. Phys. Condens. Matter*, vol. 21, no. 10, p. 104204, 2009.

[11] A. Y. Lee, C. J. Powell, J. M. Gorham, A. Morey, J. H. J. Scott, and R. J. Hanisch, "Development of the nist x-ray photoelectron spectroscopy (xps) database, version 5," *Data Science Journal*, vol. 23, no. 1, 2024.

[12] J. P. Perdew, K. Burke, and M. Ernzerhof, "Generalized gradient approximation made simple," *Phys. Rev. Lett.*, vol. 77, no. 18, pp. 3865–3868, 1996.
